# Protein arginine methyltransferases coordinate mitochondrial stress adaptation and neuromuscular function

**DOI:** 10.1038/s12276-026-01762-8

**Published:** 2026-07-03

**Authors:** Ju-Hyeon Bae, Chang-Lim You, Jeongmin Park, Jong-Sun Kang

**Affiliations:** 1 Department of Molecular Cell Biology, Sungkyunkwan University, Suwon, Republic of Korea; 2 Department of Metabiohealth, Sungkyunkwan University, Suwon, Republic of Korea

**Keywords:** Molecular biology, Cell biology

## Abstract

Sarcopenia and neuromuscular degeneration are key drivers of functional decline during ageing and arise not solely from muscle loss but also from failure of mitochondrial and metabolic stress adaptation across the neuromuscular system. Mitochondrial dysfunction, characterized by impaired oxidative phosphorylation, defective quality control and redox imbalance, contributes directly to muscle weakness, neuromuscular junction instability and motor unit degeneration. However, the upstream mechanisms governing the transition from adaptive remodelling to degenerative collapse remain incompletely defined. Protein arginine methyltransferases (PRMTs) have emerged as critical modulators of mitochondrial and metabolic stress signalling. Beyond epigenetic regulation, PRMTs influence signalling pathways that intersect with AMP-activated protein kinase (AMPK)–Forkhead box O (FOXO) and mechanistic target of rapamycin (mTOR), thereby regulating mitochondrial biogenesis, selective autophagy and mitophagy, proteostatic balance, and anabolic restraint. Distinct PRMT family members exert non-redundant functions across muscle fibres, satellite cells and motor neurons, collectively shaping neuromuscular stress resilience. We propose that PRMTs act as molecular rheostats that bias cellular responses to mitochondrial stress towards adaptive resolution or progression to neuromuscular degeneration, thereby positioning PRMT-regulated metabolic signalling as a unifying mechanism underlying sarcopenia and compromised healthspan.

## PRMTs shape muscle resilience against ageing and degeneration

As life expectancy rises, maintaining functional independence and physiological resilience becomes crucial, with skeletal muscle playing a central role in healthspan by supporting movement, metabolism and stress adaptation. This Review highlights mitochondrial dysfunction as a key driver of muscle ageing and neuromuscular decline, extending beyond muscle mass to impair strength and endurance. Protein arginine methyltransferases (PRMTs) are presented as critical regulators of mitochondrial quality and stress signalling, modulating pathways such as AMP-activated protein kinase (AMPK) and mechanistic target of rapamycin (mTOR). PRMTs coordinate mitochondrial biogenesis, autophagy and mitophagy, thereby influencing muscle functional resilience. Distinct PRMTs act across muscle fibres and motor neurons, with PRMT1 promoting mitochondrial biogenesis and PRMT4 regulating mitochondrial fission. Targeting PRMT-dependent pathways may restore mitochondrial adaptability and provide therapeutic opportunities for sarcopenia and neuromuscular diseases, enhancing systemic metabolic resilience during ageing. This summary was initially drafted using artificial intelligence, then revised and fact-checked by the author.

## Introduction

As life expectancy continues to increase, preservation of functional independence and physiological resilience, rather than lifespan alone, has emerged as a central biomedical objective. Skeletal muscle is a major determinant of healthspan, governing locomotion, metabolic homeostasis and systemic stress tolerance^1,2^. Age-associated deterioration of muscle mass, strength and contractile performance culminates in sarcopenia, a condition strongly associated with frailty, falls, insulin resistance and increased mortality^3^. While reduced physical activity, endocrine alterations and nutritional insufficiency contribute to sarcopenia, these factors do not fully account for the early and progressive decline in muscle function observed with ageing^4,5^.

Accumulating evidence identifies mitochondrial and metabolic dysfunction as central drivers of sarcopenia and neuromuscular decline^6,7^. Ageing muscle exhibits reduced oxidative phosphorylation efficiency, impaired redox balance and defective mitochondrial quality control, abnormalities that precede overt muscle loss and correlate more strongly with weakness and fatigability than muscle mass itself^8–10^. Importantly, mitochondrial dysfunction extends beyond myofibres to motor neurons and neuromuscular junctions (NMJs), implicating coordinated failure of neuromuscular bioenergetics in functional decline^11–13^.

Mitochondrial stress responses are tightly coupled to metabolic signalling pathways that sense energetic and nutrient status, particularly AMP-activated protein kinase (AMPK), Forkhead box O (FOXO) transcription factors and mechanistic target of rapamycin (mTOR)^14,15^. This signalling triad governs mitochondrial biogenesis, autophagy and mitophagy, proteostasis, and anabolic restraint^16–18^. Dysregulation of these pathways shifts mitochondrial stress responses from adaptive remodelling toward bioenergetic failure and degeneration.

Within this regulatory landscape, protein arginine methyltransferases (PRMTs) have emerged as critical modulators of metabolic and stress signalling. PRMTs catalyse arginine methylation of histone and non-histone substrates, thereby regulating protein–protein interactions, subcellular localization, stability and transcriptional output^19,20^. Although historically studied as epigenetic regulators, PRMTs are now recognized as broad signalling integrators that modify transcriptional coactivators, transcription factors, kinases, RNA-binding proteins and stress-response machinery^21–23^.

Importantly, PRMTs do not function as primary metabolic sensors. Instead, they tune the amplitude, duration and context of canonical stress pathways, including AMPK, FOXO and mTOR signalling^24–28^. Through this modulatory role, PRMTs act as molecular rheostats that determine whether mitochondrial and metabolic stress elicits adaptive renewal, such as mitochondrial biogenesis and selective autophagy, or progresses toward maladaptive outcomes, including oxidative damage, excessive catabolism, NMJ instability and neuromuscular degeneration. In this Review, we incorporate current evidence to propose that dysregulation of PRMT-regulated metabolic stress signalling represents a key upstream mechanism driving mitochondrial dysfunction, sarcopenic progression and loss of neuromuscular healthspan.

## Mitochondrial dysfunction as a central driver of muscle ageing and neuromuscular degeneration

Mitochondria serve as the primary metabolic hubs of skeletal muscle, integrating ATP production, substrate utilization, redox signalling and adaptive stress responses^3,29^. With ageing, muscle mitochondria exhibit reduced respiratory efficiency, altered fission–fusion balance, impaired mitochondrial turnover, and accumulation of mitochondrial DNA and protein damage^30,31^. These defects compromise energy supply whilst increasing reactive oxygen species (ROS) production, directly linking bioenergetic insufficiency to oxidative stress.

Crucially, mitochondrial dysfunction is not merely a consequence of muscle loss but also an early and causative event in neuromuscular ageing^6,32^. Declines in mitochondrial respiratory capacity and coupling efficiency correlate more closely with muscle weakness and fatigability than with muscle mass alone^8,33^. This indicates that age-related declines in muscle strength and contractile performance can occur independently of muscle size, a phenomenon referred to as dynapenia, which is distinct from sarcopenia and is closely linked to mitochondrial dysfunction^34,35^. Excess mitochondrial ROS damages contractile proteins, calcium-handling machinery and signalling components, reinforcing a feed-forward cycle of functional decline^36^. Consistent with this, skeletal muscle-specific deletion of MnSOD (SOD2) increases mitochondrial ROS, impairing contractile force, endurance and calcium handling, and causing NMJ fragmentation, all without reducing muscle mass^37^.

Mitochondrial impairment also disrupts intracellular signalling pathways governing proteostasis, inflammation and regeneration^38–40^. Defective autophagy and mitophagy permit accumulation of dysfunctional organelles, overwhelming protein quality control systems and destabilizing myofibre architecture^11,41–45^. These changes sensitize muscle to FOXO-driven catabolic signalling and limit adaptive remodelling under stress.

**Fig. 1:**
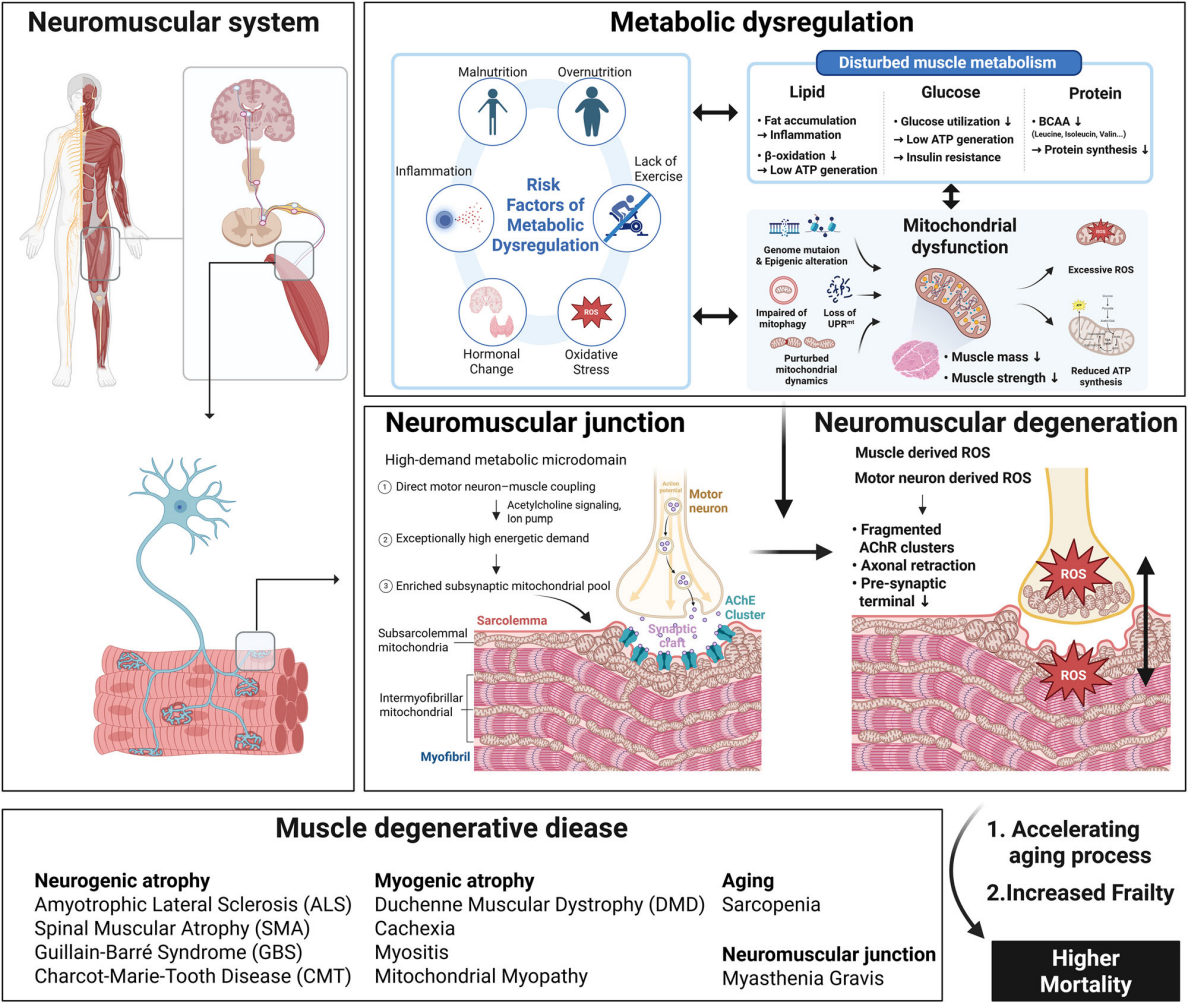
The metabolic–mitochondrial–neuromuscular axis exacerbates muscle degeneration. The neuromuscular system is an integrated network comprising the brain, spinal cord, motor neurons, neuromuscular junctions and skeletal muscle, in which muscle actively communicates with the nervous system rather than serving as a passive target. Systemic homeostasis depends on sustained cellular energy supply and effective oxidative stress control, both of which rely on intact metabolic function and mitochondrial integrity. Nutritional imbalance, physical inactivity and chronic inflammation disrupt metabolic homeostasis, leading to lipid accumulation, impaired glucose metabolism, reduced protein synthesis and decreased availability of branched-chain amino acids (BCAAs). These metabolic disturbances compromise mitochondrial quality control, resulting in reduced ATP production and excessive reactive oxygen species (ROS) generation. The ensuing bioenergetic failure and oxidative stress destabilize muscle structure and neuromuscular signalling. Neuromuscular junctions, which are highly enriched in mitochondria, are particularly vulnerable to defects in mitochondrial quality control, with dysfunction propagating between muscle fibres and motor neurons. This pathological cascade accelerates degenerative muscle disorders, including motor neuron diseases, myopathies, vulnerability, disease severity and patient mortality. UPR, unfolded protein response. This figure was generated using Biorender.com.

Importantly, mitochondrial dysfunction in muscle not only affects the muscle itself but also extends to the NMJ and motor neurons. Crucially, these effects are not restricted to motor neuron, Schwann cell-specific mitochondrial dysfunction models, and muscle-specific mitochondrial defect models provide compelling evidence that primary mitochondrial dysfunction in muscle is sufficient to destabilize the NMJ and induce secondary neuronal pathology, underscoring the bidirectional nature of neuromuscular degeneration (Table 1). Skeletal muscle mitochondria form a specialized network with spatially distinct subpopulations: subsarcolemmal mitochondria beneath the plasma membrane contribute to local ATP supply and participate in Ca²⁺, insulin, and IGF signalling and ROS sensing, whereas intermyofibrillar mitochondria primarily support ATP production for muscle contraction through oxidative phosphorylation^46^. Of note, mitochondria positioned beneath the NMJ (subsynaptic mitochondria) represent a specialized subsarcolemmal mitochondrial subpopulation, exhibiting the highest mitochondrial density within the fibre to meet the substantial energy demands of acetylcholine signalling and ion pump activity, and are particularly vulnerable due to their high local ATP demand and strict redox requirements^47,48^. Local mitochondrial failure compromises synaptic maintenance and transmission, reducing the reliability of neuromuscular transmission and predisposing NMJs to fragmentation and denervation. Reduced neuromuscular activity further exacerbates mitochondrial stress within muscle fibres^12,49,50^, establishing a bidirectional mitochondria–neuromuscular axis in which metabolic failure and neuromuscular instability amplify one another during ageing (Fig. 1).

**Table 1:** Phenotypes and mechanisms of tissue-specific mitochondrial defect models in myofibres, motor neurons and Schwann cells.

Genetically modified region	Mouse model	Key phenotype	Mechanism	Refs.
Myofibre specific	*Prmt1* knockout (*Prmt1*^*fl/fl*^; *Myl1-Cre*)	Skeletal muscle atrophy Reduced muscle strength and endurance Loss of type 2 myofibres Accumulation of dysfunctional mitochondria Accumulation of dysfunctional mitochondria Increased oxidative stress Impaired mitochondrial respiration Defective mitophagy NMJ fragmentation Reduced NMJ stability Partial denervation	Impaired mitophagy pathway (TBK1–OPTN)	^12^
	*Atg7* knockout (*Atg7*^*fl/fl*^*; MCK-Cre*)	Skeletal muscle atrophy Reduced muscle strength and endurance Loss of type 2 myofibres Accumulation of dysfunctional mitochondria Increased oxidative stress Impaired autophagic flux Premature ageing-like muscle phenotype NMJ fragmentation Partial denervation Reduced reinnervation capacity	Autophagy failure	^11^
	*Drp1* knockout (*Drp1* ^*fl/fl*^*; HSA-Cre*)	Skeletal muscle atrophy Reduced muscle strength, endurance and exercise capacity Hyperfused and enlarged mitochondrial network Impaired mitochondrial respiration Defective mitochondrial Ca²⁺ handling and excitation–contraction coupling NMJ fragmentation Partial denervation	Mitochondrial fission failure	^126^
	*Opa1* knockout (*Opa1* ^*fl/fl*^*; MCK-Cre*)	Skeletal muscle atrophy Reduced muscle strength Mitochondrial content reduction Mitochondrial depolarization Impaired mitochondrial respiration Activation of unfolded protein response/endoplasmic reticulum stress Accumulation of dysfunctional mitochondria Partial denervation Impaired innervation	Mitochondrial fragmentation	^127^
	*Raptor* knockout (*Raptor* ^*fl/fl*^*; HSA-Cre*) *mTOR* knockout (*mTOR* ^*fl/fl*^*; HSA-Cre*)	Skeletal muscle atrophy Reduced muscle strength Myofibre type shift Impaired protein synthesis capacity Mitochondrial depolarization Impaired autophagic flux NMJ fragmentation Partial denervation Impaired synaptic transmission Reduced NMJ stability	Blocking of mTORC1 signalling	^128^
	*Tsc1* knockout (*Tsc1*^*fl/fl*^*; HSA-Cre*)	Skeletal muscle atrophy Suppressed autohagic flux Reduced NMJ stability Increased neuromuscular transmission fatigability Exacerbated skeletal muscle atrophy after denervation	Hyperactivation of mTORC1 signalling	^13^
	*Hdac4* knockout (*Hdac4*^*fl/fl*^*; Myog-Cre*)	Skeletal muscle atrophy Body weight loss Partial denervation Reduction in synapse area and NMJ fragmentation NMJ functional deficit Lack of ubiquitin–proteasome system activation Impaired autophagic flux	Denervation-responsive transcriptional programme	^129^
	*4Ebp1* mutant (*MCK-Cre*)	Acetylcholine receptor cluster fragmentation Enhanced NMJ synaptic transmission functions Induced severe NMJ remodelling Increased number of post-synaptic myonuclei Activation of muscle regeneration Skeletal muscle atrophy with enhanced mitochondrial function and basal autophagy Expansion of endplate area Increased neuromuscular transmission fatigability	Reduced cap-dependent translation (eIF4E)	^130^
Motor neuron specific	*Cdo* knockout (*Cdo*^*fl/fl*^*; Hb9-Cre*)	NMJ instability and partial denervation Distal motor axon degeneration Skeletal muscle atrophy Impaired neuromuscular connectivity and motor function Dying-back neurodegenerative phenotype	Impaired neurotrophin receptor-mediated signalling (NRG1–ErbB–Akt axis)	^131^
	*Prmt1* knockout (*Prmt1*^*fl/fl*^; *Hb9-Cre*)	Motor neuron degeneration Skeletal muscle atrophy Impaired nerve injury recovery NMJ structural abnormalities Elevated cellular stress response Mitochondrial dysfunction	PRMT1-dependent mitochondrial maintenance and stress-response pathway	^84^
	*Tardbp* knockout (*Tardbp* ^*fl/fl*^; *VAChT-Cre*)	Progressive motor dysfunction and weight loss Large motor axon degeneration Partial denervation Skeletal muscle atrophy Delayed motor neuron soma atrophy Ultra-structural autophagy abnormalities	RNA metabolism dysregulation Altered autophagy-related structures	^132^
	*Atg7* knockout (*Atg7*^*fl/fl*^; *ChAT-Cre*)	Early NMJ instability and structural abnormalities Distal motor axon degeneration Autophagy substrates accumulation Skeletal muscle atrophy with impaired motor performance Increased neurodegenerative vulnerability	Defective autophagy–lysosome pathway	^133^
	*Tbk1* knock-in (ALS-associated *Tbk1* mutation)	Partial denervation Distal motor axon degeneration Early-onset motor dysfunction and reduced motor performance Mitochondrial and proteostatic stress Increased neurodegenerative vulnerability	Impaired mitophagy and mitochondrial energy homeostasis (TBK1–OPTN)	^134^
	*Fus* mutant (*ChAT-Cre*)	Progressive motor dysfunction Partial denervation Distal motor axon degeneration Motor neuron degeneration Skeletal muscle atrophy	RNA granule dysfunction	^135^
	*Srf* knockout in ALS model (*ChAT-Cre; SOD1*^*G93A*^)	Earlier disease onset Worsened motor performance at early disease stages Increased NMJ degeneration Increased neuroinflammation Motor neuron number and survival only modestly affected	Activity-dependent transcription pathway	^136^
	*Sod1*-inducible knockout (*i-mnSod1*KO)	Partial denervation Motor axon calibre shift Simplified and fragmented homology Skeletal muscle atrophy Progressive motor weakness	Oxidative stress defence failure	^137^
	*Syt13* heterozygous mutant (*Syt13*^*+/−*^)	ALS/spinal muscular atrophy-like neurodegeneration Synaptic contact loss Protein aggregation features Motor neuron vulnerability Reduced NMJ function	Presynaptic vesicle maintenance pathway	^138^
Schwann cell specific	*c-Jun* knockout (*c-Jun* ^*fl/fl*^; *P0-Cre*)	Impaired nerve regeneration Failure of functional recovery Increased neuronal death after injury Loss of Schwann cell identity Prolonged denervation-associated pathology	Repair Schwann cell transcriptional programme	^139^
	*Sox10* knockout (*Sox10*^*fl/fl*^; *Dhh-Cre*)	Loss of Schwann cell identity Loss of Schwann cell identity Abnormal myelination Peripheral nerve dysfunction Secondary axonal pathology	Schwann cell lineage maintenance	^140^
	*Lkb1* knockout (*Lkb1*^*fl/fl*^; *P0-Cre*)	Impaired Schwann cell-mediated axon maintenance Progressive axon degeneration Peripheral neuropathy phenotype Peripheral nerve dysfunction Motor impairment	Schwann cell metabolic/axon-support signalling	^141^
	*Atg7* knockout (*Atg7*^*fl/fl*^; *P0-Cre*)	Abnormal myelinating Schwann cell cytoplasm remodelling Abnormal myelination Peripheral nerve structural abnormalities and dysfunction Vulnerability to demyelinating stress	Schwann cell autophagy	^142^
	*Hgs* knockout (*Hgs*^*fl/fl*^; *P0-Cre*)	Abnormal myelination Peripheral nerve dysfunction Secondary axonal degeneration Motor impairment	Endosomal sorting and receptor trafficking	^143^
	*Pi4ka* knockout (*Pi4ka*^*fl/fl*^; *P0-Cre*)	Abnormal myelination Impaired nerve conductivity Motor functional deficits Progressive neuropathy features peripheral nervous system structural defects	Phosphoinositide synthesis for myelin growth	^144^
	*CnB* knockout (*CnB*^*fl/fl*^; *P0-Cre*)	Normal developmental myelination largely preserved Delayed myelin clearance after injury Reduced autophagy activation after crush Impaired repair milieu Slower functional recovery	Injury-induced autophagy regulation (calcineurin–TFEB axis)	^145^

*ALS* amyotrophic lateral sclerosis, *mTORC1* mechanistic target of rapamycin complex 1, *NMJ* neuromuscular junction.

## PRMT-dependent regulation of mitochondrial plasticity and metabolic stress signalling

### PRMTs as integrators of mitochondrial quality control and metabolic signalling

**Fig. 2:**
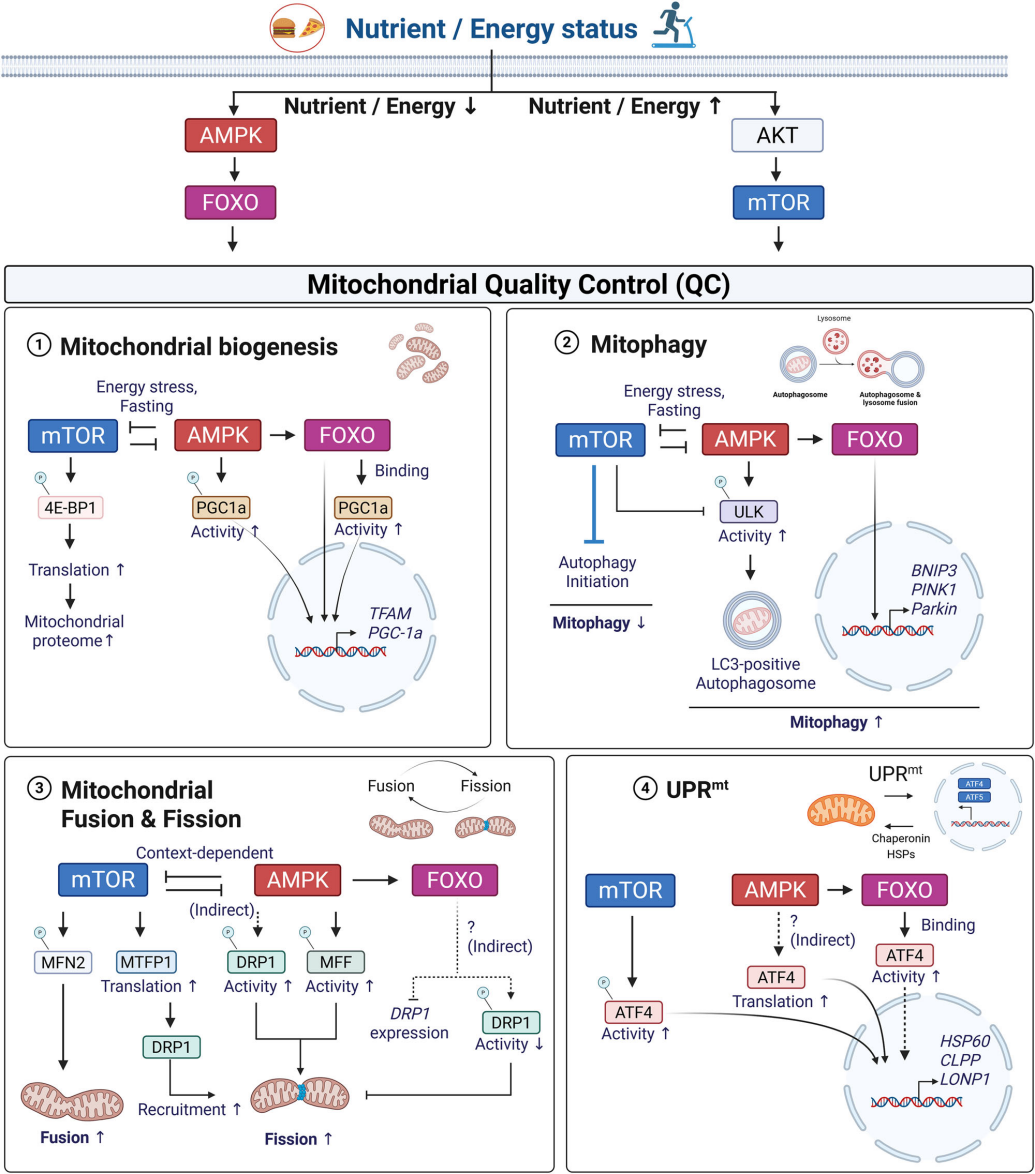
Metabolic stress-responsive mTOR–AMPK–FOXO axis in mitochondrial quality control. Nutrient and energy status regulate the mechanistic target of rapamycin (mTOR)–AMP-activated protein kinase (AMPK)–Forkhead box O (FOXO) axis, with low energy activating AMP–FOXO-driven catabolic signalling and nutrient sufficiency engaging mTOR-dependent anabolic programmes, together coordinating mitochondrial quality control. AMPK phosphorylates PGC1α to enhance its transcriptional activity and induce downstream TFAM, while FOXO further promotes PGC1α expression. In contrast, mTOR phosphorylates 4EBP1 to stimulate translation of mitochondria-associated proteins. Mitophagy is promoted by AMPK and FOXO but suppressed by mTOR. AMPK phosphorylates ULK1 at Ser317 and Ser777 to initiate autophagy, whereas mTORC1 phosphorylates ULK1 at Ser757, preventing AMPK interaction and inhibiting autophagy initiation. Mitochondrial dynamics are context-dependent: mTOR can promote fusion via MFN2 or, under stress, drive fission through MTFP-mediated DRP1 activation; AMPK similarly promotes fission via DRP1, while FOXO may influence fission indirectly. Mitochondrial unfolded protein response (UPR^mt^) signalling is mediated by ATF4, with mTOR enhancing ATF4 activity via phosphorylation, AMPK increasing ATF4 translation, and FOXO interacting with ATF4 to further amplify its transcriptional activity. This figure was generated using Biorender.com.

Mitochondrial homeostasis is maintained through coordinated regulation of mitochondrial biogenesis, dynamics (fusion and fission), unfolded protein response (UPR^mt^) and selective turnover (mitophagy), which together determine mitochondrial quality rather than abundance alone^51,52^. These processes are tightly integrated with metabolic signalling through the AMPK–FOXO–mTOR axis, which senses energetic stress, oxidative damage and nutrient availability to balance renewal, degradation, and growth^13–15^. Low-energy states activate AMPK–FOXO-driven catabolic programmes, whereas nutrient sufficiency engages mTOR-dependent anabolic signalling, with both arms directly modulating mitochondrial quality control^14,15^ (Fig. 2).

PGC1α, a master regulator of mitochondrial biogenesis, is phosphorylated by AMPK, enhancing its transcriptional activity^53–55^. AMPK also activates FOXO, which further induces PGC1α expression through direct binding or promoter engagement, leading to downstream induction of TFAM and mitochondrial gene transcription^56–58^. In contrast, mTOR promotes mitochondrial biogenesis by phosphorylating 4EBP1, releasing eIF4E and enhancing translation of mitochondria-related proteins^59^.

Mitophagy is primarily stimulated by AMPK and FOXO, whereas mTOR acts as a negative regulator. AMPK phosphorylates ULK1 at Ser317 and Ser777, initiating LC3-dependent autophagosome formation and mitophagy^60^. Conversely, mTORC1 phosphorylates ULK1 at Ser757, preventing its interaction with AMPK and suppressing autophagy initiation^61^. FOXO transcription factors upregulate core autophagy genes and selectively induce mitophagy-specific genes, including PINK1, Parkin and BNIP3 (refs. ^62–64^).

Mitochondrial dynamics regulated by mTOR, AMPK and FOXO are highly context-dependent. mTOR can promote mitochondrial fusion via MFN2 activation but, under stress conditions such as amyloid-β exposure, it enhances MTFP1 translation, driving DRP1 phosphorylation and fission. AMPK similarly promotes fission through DRP1 activation^65–68^. Although FOXO factors do not directly control fusion–fission gene expression, FOXO1 inhibition correlates with decreased DRP1 expression and Ser616 phosphorylation, suggesting an indirect regulatory role^69^.

**Fig. 3:**
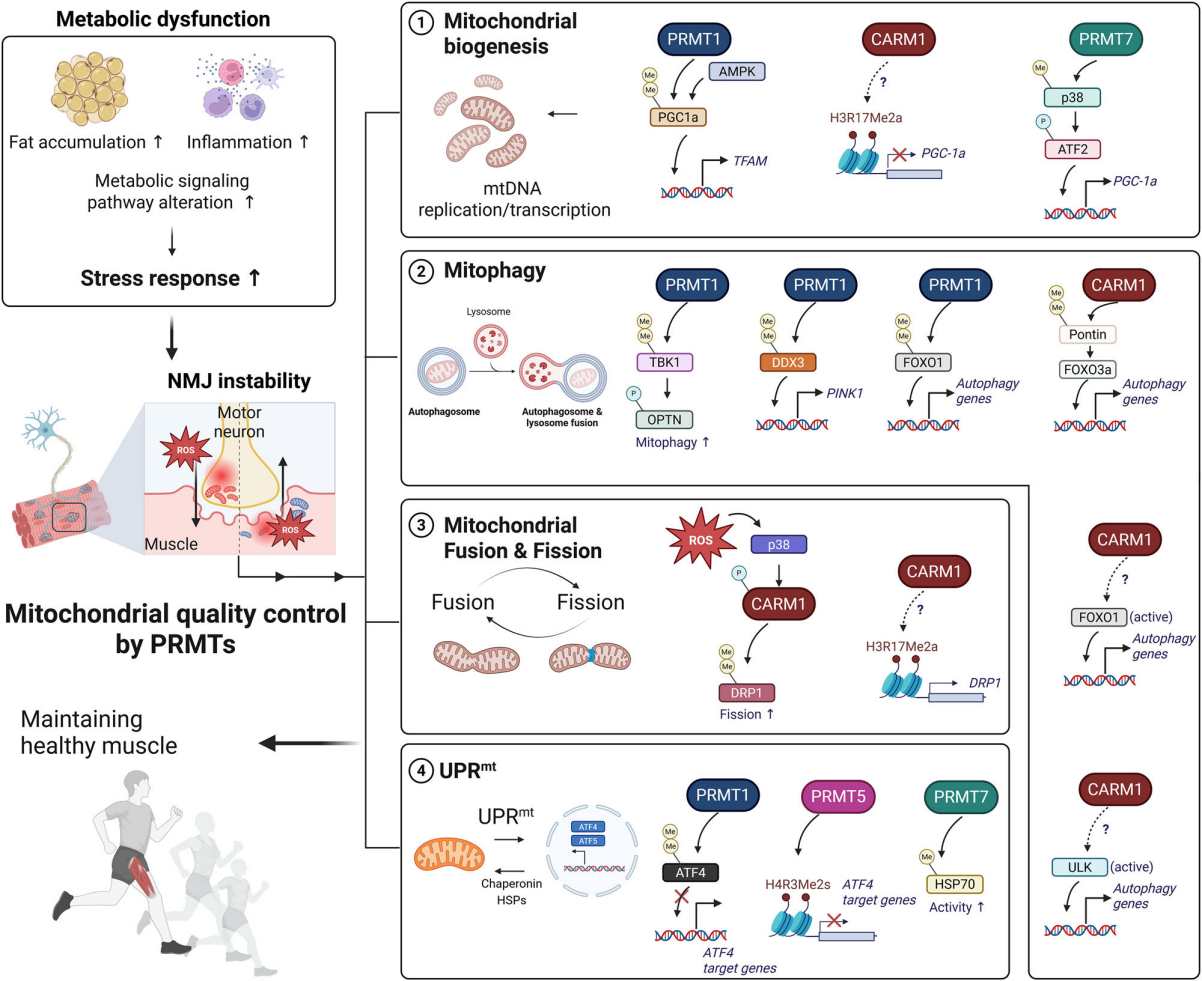
Differential roles of PRMT isoforms in mitochondrial homeostasis. Protein arginine methyltransferase (PRMT) family members act as key regulators linking mitochondrial homeostasis to cellular stress responses through isoform-specific mechanisms. Metabolic dysfunction driven by lipid accumulation, chronic inflammation and dysregulated signalling imposes cellular stress that disrupts mitochondrial function in the neuromuscular system. PRMT enzymes mitigate these defects by regulating mitochondrial quality control at both epigenetic and non-epigenetic levels. PRMT1 and PRMT7 enhance PGC1α expression to promote mitochondrial biogenesis, whereas CARM1 has been reported to suppress biogenesis. Mitophagy is primarily regulated by PRMT1 and CARM1, with CARM1 additionally promoting DRP1 expression and activation under oxidative stress. In the context of mitochondrial unfolded protein response (UPR^mt^), PRMT1 and PRMT5 suppress ATF4 expression and transcriptional activity, while PRMT7 enhances HSP70 activity, indicating isoform-specific roles of PRMTs in coordinating mitochondrial stress responses. This figure was generated using Biorender.com.

UPR^mt^ signalling is largely mediated by ATF4. mTOR directly phosphorylates ATF4 to promote expression of UPR^mt^ genes^70^, while AMPK activation enhances ATF4 translation without direct phosphorylation^71^. FOXO transcription factors interact with ATF4 to amplify its transcriptional activity, though direct FOXO-dependent activation of UPR^mt^ genes remains unclear^72^. PRMT-dependent arginine methylation intersects this network, coordinating mitochondrial plasticity with metabolic state rather than acting on isolated quality-control pathways (Fig. 3).

Collectively, AMPK promotes mitochondrial biogenesis and initiates autophagy and mitophagy, FOXO coordinates stress-adaptive catabolic programmes, and mTOR integrates nutrient cues to drive anabolic growth while restraining autophagic flux^16–18^. PRMTs fine-tune these pathways by modulating transcriptional output, signalling intensity and pathway crosstalk.

To date, nine PRMT isoforms (PRMT1–9) have been identified in mammals, and they are broadly classified into three types based on the methylarginine products they generate (Table 2). Type I PRMTs, including PRMT1, PRMT2, PRMT3, CARM1 (PRMT4), PRMT6 and PRMT8, catalyse the formation of asymmetric dimethylarginine (ADMA). Type II PRMTs, represented by PRMT5 and PRMT9, generate symmetric dimethylarginine (SDMA). Type III PRMTs, with PRMT7 as the only known member, produce monomethylarginine. These distinct forms of arginine methylation differentially regulate protein function and gene expression, thereby playing critical roles in cellular stress responses.

**Table 2:** Overview of PRMT family members, including key substrates, tissue specificity and functional outcomes.

PRMT family member	Key substrates		Tissue specificity (based on GTEx)	Functional outcomes
	Histone	Non-histone		
PRMT1	H3R3, H4R3, H2AR11	PGC1α, TBK1, FOXO1, P53, DDX3, NF-kb 65, ATF4, RNA-binding protein	Expressed in almost all tissues	Transcriptional activation, DNA repair, signal transduction
PRMT2	H3R8	Oestrogen receptor (ERα), RB	Generally low expression Higher in liver and heart	Gene regulation, modulation of hormone signalling
PRMT3	H4	RPS2	Mainly expressed in liver and pancreas	Ribosome biogenesis, protein synthesis
PRMT4 (CARM1)	H3R2, H3R17, H3R26, H3R42	CBP/p300 coactivators, DRP1, pontin	Tissue-specific expression (high in testis and brain, low in muscle)	Transcriptional activation, cell differentiation
PRMT5	H2AR3, H3R2, H3R8, H4R3	Sm proteins	Expressed in almost all tissues	Transcriptional repression, RNA splicing, cell cycle control
PRMT6	H3R2, H2AR11, H2AR29	FOXO3a, DNA polymerase-β	Limited expression Higher in liver and lung	Transcriptional repression, DNA repair
PRMT7	H3R2, H4R3, H4R17	eIF2α, p38 MAPK, HSP70, PGC1α	Tissue-specific expression (high in testis, moderate in muscle and heart, low in brain)	Stress response, stem cell maintenance
PRMT8	–	SOX2, g-H2AX	Mostly brain-specific expression	Neural development, synaptic function
PRMT9	–	SAP145 (splicing factor)	Limited, expressed in a few tissues (liver)	RNA splicing, gene expression regulation

*PRMTs* protein arginine methyltransferases.

In this section, we first outline core mitochondrial quality control pathways, followed by PRMT-specific regulatory roles (PRMT1, CARM1, PRMT5, PRMT7 and PRMT8), with particular emphasis on distinguishing direct versus indirect mechanisms. Notably, PRMTs regulate autophagy primarily at the level of upstream stress integration, particularly through FOXO-dependent transcription and AMPK-linked autophagic competence, thereby preventing both inadequate mitochondrial clearance and excessive, degenerative autophagy^24–26^. Through this balanced control, PRMTs determine whether mitochondrial stress resolves through adaptive renewal or progresses toward oxidative damage, bioenergetic failure, and neuromuscular degeneration.

#### PRMT1

PRMT1 functions as a central amplifier of mitochondrial biogenesis and mitophagy by integrating metabolic stress signalling in skeletal muscle. Among the PRMT family members, PRMT1 is the most abundant and extensively studied isoform in skeletal muscle and has emerged as a central regulator of mitochondrial plasticity and stress adaptation^26^. PRMT1 positively regulates mitochondrial biogenesis primarily through modulation of PGC1α-dependent transcriptional programmes. Direct arginine methylation of PGC1α enhances its coactivator activity, promoting expression of genes involved in oxidative phosphorylation, mitochondrial respiration and fatty acid metabolism^7,73^. In this context, PRMT1 amplifies PGC1α signalling downstream of AMPK activation, stabilizing oxidative gene programmes and converting transient energetic stress into durable mitochondrial remodelling. Consistent with this role, PRMT1 expression and activity are increased in response to metabolic stress, exercise and denervation, positioning PRMT1 as an amplifier of adaptive mitochondrial remodelling in muscle^12,25,74^.

Beyond mitochondrial biogenesis, PRMT1 plays an important role in mitochondrial quality control by integrating stress-responsive transcription with selective mitophagy. Under metabolic and oxidative stress, PRMT1 can enhance FOXO transcriptional activity, promoting expression of genes involved in antioxidant defence, autophagy and mitochondrial surveillance^18,24^. When coupled to effective mitochondrial clearance, this FOXO-dependent response supports adaptive remodelling; however, sustained FOXO activation in the context of impaired mitophagy becomes maladaptive, driving excessive proteolysis and mitochondrial depletion. Thus, PRMT1 functions as a gain controller of FOXO output rather than as a simple activator, tuning the balance between stress adaptation and degenerative catabolism.

In parallel, PRMT1 directly regulates selective mitophagy through TBK1–OPTN-dependent signalling, a pathway essential for efficient removal of damaged mitochondria under metabolic and inflammatory stress^12^. PRMT1 mediates methylation of TBK1 at R54, R134 and R228, and this asymmetric arginine modification is essential for TBK1 activation^75^. Through arginine methylation-dependent regulation of signalling components, such as TBK1, PRMT1 fine-tunes mitophagic flux, preventing accumulation of dysfunctional mitochondria that would otherwise impair bioenergetic efficiency and increase oxidative stress. Disruption of PRMT1 activity in skeletal muscle results in mitochondrial abnormalities, defective mitophagy and elevated oxidative stress, phenotypes that precede overt muscle atrophy. These mitochondrial defects are accompanied by NMJ instability and denervation-like features, highlighting a functional link between PRMT1-dependent mitochondrial surveillance and neuromuscular integrity^12^. Additionally, another study, conducted in cancer cells, reported that PRMT1 enhances DDX3 stability via arginine methylation, thereby promoting PINK1 expression and mitophagy, and proposed this mechanism as a molecular basis for mitochondrial function regulation^76^.

In cardiac muscle studies, PRMT1 has been shown to attenuate excessive endoplasmic reticulum stress by promoting ATF4 methylation^77^. While a direct role in the UPR^mt^ was not examined, given that ATF4 is a key transcriptional regulator of UPR^mt^, these findings raise the possibility that PRMT1 may indirectly influence UPR^mt^ activity through broader regulation of ATF4-dependent stress signalling.

Collectively, these findings position PRMT1 as a key integrator of multiple pathways influencing mitochondrial and cellular homeostasis, including PGC1α-mediated mitochondrial biogenesis, FOXO-dependent stress responses, PRMT1–DDX3-facilitated PINK1-linked mitophagy and PRMT1-mediated TBK1 activation; however, while PRMT1 interacts with and modulates several signalling components, a direct role for PRMT1 in activating AMPK per se has not been conclusively established. Dysregulation of PRMT1 shifts the balance from adaptive mitochondrial remodelling toward bioenergetic failure, oxidative stress and neuromuscular deterioration, positioning PRMT1 as a critical determinant of mitochondrial and neuromuscular homeostasis during ageing.

#### CARM1 (PRMT4)

CARM1 primarily regulates mitochondrial dynamics by promoting stress-induced fission and constraining mitochondrial expansion. CARM1 regulates mitochondrial homeostasis by coupling transcriptional control with post-translational regulation of mitochondrial dynamics, thereby shaping mitochondrial morphology, turnover and stress responsiveness in a context-dependent manner^78–80^. Unlike PRMT1, which primarily enhances mitochondrial biogenesis and mitophagy, CARM1 functions to constrain mitochondrial expansion while tuning dynamic remodelling and quality control^78^.

In the nucleus, CARM1 modulates mitochondrial homeostasis through transcriptional repression of mitochondrial biogenesis programmes^78^. By downregulating PGC1α and TFAM while promoting expression of DNM1L (DRP1), CARM1 limits mitochondrial expansion and biases mitochondrial networks toward fission. This nuclear activity positions CARM1 as a regulator that restrains mitochondrial content while maintaining dynamic flexibility under basal and metabolic stress conditions.

Under oxidative stress, CARM1 undergoes phosphorylation-dependent translocation to the cytoplasm, where it directly methylates DRP1, accelerating mitochondrial fission^79^. This post-translational regulation amplifies mitochondrial fragmentation and increases mitochondrial ROS production, reinforcing redox signalling. Sustained cytoplasmic CARM1 activity establishes a positive feedback loop between oxidative stress and mitochondrial fission, promoting bioenergetic inefficiency and stress-induced functional decline.

Importantly, CARM1 also supports basal autophagic and mitophagic competence in coordination with metabolic signalling. Through regulation of AMPK–ULK1 and AKT–FOXO pathways, CARM1 helps ensure that mitochondrial fission is coupled to efficient turnover rather than unchecked fragmentation^80^. Loss of CARM1 disrupts this coordination, leading to impaired autophagic flux, accumulation of dysfunctional mitochondria and defective adaptive remodelling despite reduced mitochondrial biogenesis^80^. Although not conducted in muscle, this study demonstrated that CARM1-mediated arginine methylation of pontin promotes FOXO3a-dependent transcription of autophagy genes, establishing an epigenetic mechanism that enhances autophagic flux^81^.

Collectively, CARM1 functions as a regulator of mitochondrial dynamic balance, tuning the relationship between biogenesis, fission and mitophagy in response to metabolic and oxidative stress. Dysregulation of CARM1 shifts mitochondrial networks toward excessive fragmentation and oxidative burden, particularly when fission outpaces mitochondrial clearance, thereby linking altered mitochondrial dynamics to bioenergetic failure and neuromuscular vulnerability during ageing.

#### PRMT5

PRMT5 maintains metabolic and regenerative competence by restraining excessive FOXO-driven catabolic signalling rather than directly regulating mitochondrial quality control. PRMT5 contributes to mitochondrial and neuromuscular homeostasis primarily by preserving regenerative and metabolic competence rather than by directly regulating mitochondrial dynamics^82,83^. Although PRMT5 is classically associated with transcriptional repression and oncogenic processes, accumulating evidence highlights an essential role in skeletal muscle progenitor maintenance and stress resilience^82,83^.

Genetic ablation of PRMT5 in embryonic myoblasts results in progressive muscle atrophy, impaired satellite cell maintenance and premature lethality despite normal embryonic development^82^. Mechanistically, PRMT5 methylates FOXO1, promoting its destabilization and restraining excessive FOXO-driven autophagy and lipophagy^82^. Loss of PRMT5 increases total FOXO1 levels and favours its cytoplasmic accumulation, leading to premature and dysregulated autophagy, depletion of lipid droplets and impaired metabolic support in myoblasts. This imbalance compromises proliferative capacity, accelerates differentiation and restricts postnatal regenerative potential.

Importantly, systemic inhibition of autophagy partially rescues lipid storage and muscle regeneration in PRMT5-deficient mice, underscoring the requirement for PRMT5-mediated restraint of FOXO-driven autophagy during muscle development and repair^83^. By preserving lipid reserves and metabolic flexibility, PRMT5 indirectly supports mitochondrial function during periods of high energetic demand, for example, during regeneration following injury or denervation.

Additionally, PRMT5 plays a critical role in maintaining proper ATF4 splicing, which ensures stable protein levels of ATF4, a key transcription factor for UPR^mt^. Although a direct role of PRMT5 in UPR^mt^ has not been demonstrated, these findings suggest that PRMT5 may be important for the expression of ATF4-dependent target genes within the UPR^mt^ pathway^84^.

In the context of ageing, declining regenerative capacity is a major contributor to sarcopenia and neuromuscular instability. PRMT5-dependent regulation of progenitor cell homeostasis and FOXO activity therefore shapes the cellular context in which mitochondrial quality control operates, indirectly supporting mitochondrial function while permitting appropriate mTOR reactivation during recovery. Collectively, these findings position PRMT5 as a regulator of regenerative and metabolic competence that acts upstream of mitochondrial stress to preserve long-term neuromuscular integrity.

#### PRMT7

PRMT7 supports mitochondrial function indirectly by sustaining oxidative metabolism and stress-adaptive transcriptional programmes in skeletal muscle. PRMT7 contributes to mitochondrial homeostasis in skeletal muscle primarily through indirect regulation of oxidative and stress-adaptive transcriptional programmes rather than through the direct control of mitochondrial dynamics or core quality control machinery^85^. In contrast to PRMT1 and CARM1, which directly modulate mitochondrial biogenesis, fission and mitophagy, PRMT7 influences mitochondrial function by shaping the muscle metabolic phenotype and its transcriptional responsiveness to energetic stress.

Genetic loss of PRMT7 leads to reduced expression of PGC1α and downstream oxidative genes in skeletal muscle, resulting in a shift toward a more glycolytic muscle phenotype and diminished mitochondrial oxidative capacity^86^. These changes are accompanied by reduced endurance and impaired metabolic flexibility, indicating that PRMT7 supports mitochondrial energy metabolism by sustaining oxidative gene programmes. Mechanistically, PRMT7 has been linked to stress-activated signalling pathways, including p38 MAPK–ATF2, which regulates PGC1α transcription. Moreover, in vitro studies have shown that PRMT7 methylates arginine residues R548 and R753 in the C-terminal region of PGC1α, with methylation occurring preferentially at lower temperatures (≤30 °C). This temperature-dependent modification suggests a potential regulatory effect on PGC1α transcriptional activity, although a direct link between methylation and enhanced activation has not yet been demonstrated^87^.

In addition, PRMT7 directly supports cellular tolerance through arginine methylation of HSP70 family chaperones; inhibition or loss of PRMT7 reduces HSP70 methylation and compromises resistance to proteotoxic stress^88^. Consequently, PRMT7 deficiency manifests primarily as reduced mitochondrial metabolic fitness rather than as defects in mitochondrial morphology or mitophagy. The combined loss of oxidative capacity and HSP70-mediated stress buffering is therefore expected to increase reliance on FOXO-dependent catabolic stress responses, lowering the threshold for AMPK–FOXO engagement.

Together, these findings position PRMT7 as a permissive regulator that sets the metabolic and proteostatic baseline required for adaptive mitochondrial remodelling, complementing the more direct mitochondrial quality control functions of PRMT1 and CARM1.

#### PRMT8

PRMT8 acts as a neuron-specific regulator of mitochondrial stress tolerance and NMJ stability. PRMT8 is a neuron-enriched member of the PRMT family with predominant expression in the central nervous system, including spinal cord motor neurons, indicating a specialized role in neuronal maintenance rather than muscle-intrinsic regulation^89^. In vivo studies demonstrate that PRMT8 contributes to neuronal stress tolerance during ageing: genetic loss of PRMT8 reduces asymmetric dimethylarginine levels in postmitotic motor neurons, leading to premature NMJ destabilization and age-associated decline in muscle strength, accompanied by increased DNA damage and reduced CREB1 signalling^90^.

Mechanistically, PRMT8 has been linked to the regulation of membrane lipid homeostasis and neuronal stress adaptation. Following hypoxic stress, PRMT8-knockout mice exhibit altered membrane phospholipid composition, reduced mitochondrial stress capacity as assessed by oxygen consumption-based measurements, and elevated neuroinflammatory markers, whereas PRMT8 overexpression reverses these phenotypes^91^. In addition, PRMT8 has been reported to possess phosphatidylcholine-hydrolyzing phospholipase activity, generating choline and phosphatidic acid, with PRMT8 loss causing abnormal dendritic arborization and motor behaviour deficits. Together, these findings support a role for PRMT8 in coupling membrane lipid metabolism to mitochondrial stress resilience and inflammatory control in neurons.

Collectively, current evidence supports PRMT8 as a neuron-restricted regulator of stress tolerance that integrates arginine methylation and membrane lipid biology to maintain mitochondrial stress capacity and NMJ stability during ageing. Direct evidence linking PRMT8 to canonical mitochondrial quality control pathways remains limited, and further work will be required to define its neuronal substrates and compartment-specific functions.

#### PRMT-mediated regulation of mitochondrial plasticity: summary overview

The roles of PRMT family members in regulating mitochondrial quality control, metabolic signalling and stress adaptation are summarized in this section (Table 3). For each PRMT, we indicate the specific molecular targets, whether the regulation is direct or indirect, and the experimental models used, including muscle-intrinsic, neuron-specific and cancer cell studies. This compilation integrates findings from cellular, mouse and in vitro models, allowing comparison of how different PRMTs fine-tune mitochondrial biogenesis, dynamics, mitophagy, UPR^mt^ and stress tolerance pathways. By distinguishing results from cancer cells versus physiological or neuronal contexts, the table provides a comprehensive overview of the coordinated roles of PRMTs in maintaining mitochondrial plasticity.

**Table 3:** Roles of PRMT family members in regulating mitochondrial quality control, metabolic signalling and stress adaptation.

Mitochondrial quality control	PRMT family member	Mechanism	Direct/indirect	Experimental model	Refs.
Mitochondrial biogenesis	PRMT1	PGC1α arginine methylation	Direct	Saos-2 cells (cancer cells)	^73^
	CARM1 (PRMT4)	Transcriptional repression of PGC1α–TFAM	Indirect	10T1/2 cells	^78^
	PRMT7	PGC1α transcriptional regulation (p38 MAPK–ATF2 axis)	Indirect	C2C12 cells, skeletal muscle tissue (mouse)	^86^
	PRMT7	PGC1α R548/753 methylation	Direct (functional unclear)	HEK293T cells, recombinant protein	^87^
Mitophagy	PRMT1	FOXO1 R248/250 methylation	Direct	HEK293T cells	^24^
	PRMT1	TBK1 R54/134/228 methylation	Direct	C2C12 cells, skeletal muscle tissue (mouse)	^75^
	PRMT1	DDX3 Arginine methylation	Direct	MDA-MB-231 (cancer cells), BT549 (cancer cells), HEK293T cells	^76^
	CARM1 (PRMT4)	Pontin R333/339 methylation (required for FOXO3a transcriptional activity)	Direct	HeLa cells (cancer cells) mouse embryonic fibroblasts	^146^
	CARM1 (PRMT4)	Not defined, CARM1 deletion inhibits mitophagy	Direct	Skeletal muscle tissue	^80^
	PRMT5	FOXO1 symmetric demethylation (site undefined)	Direct	Myogenic progenitors (mouse)	^82^
Mitochondrial unfolded protein response	PRMT1	ATF4 R239 methylation	Indirect	H9C2 cells, HEK293T cells	^77^
	PRMT5	ATF4 transcript splicing	Indirect	Myogenic progenitors (human)	^84^
	PRMT7	HSP70 R496 methylation	Indirect	HEK293T cells, HCT116 cells (cancer cells)	^88^
Fusion/fission	CARM1 (PRMT4)	DRP1 R403/R634 methylation	Direct	10T1/2 cells	^79^
Indirect stress tolerance	PRMT8	Regulation of neuronal stress tolerance and mitochondrial respiratory capacity	Indirect	Motor neuron tissue (mouse)	^90^
	PRMT8	Membrane lipid homeostasis/mitochondrial stress resilience in neurons	Indirect	Central nervous system/motor neuron tissue (mouse)	^91^

*PRMT* protein arginine methyltransferase.

## PRMT-regulated metabolic stress signalling in neuromuscular pathology

Pathological neuromuscular conditions characterized by chronic metabolic stress, mitochondrial dysfunction and excessive ROS production, including muscle wasting syndromes, NMJ degeneration and motor neuron diseases, such as amyotrophic lateral sclerosis (ALS), highlight the vulnerability of mitochondrial quality control mechanisms that normally preserve neuromuscular integrity^92^. Emerging evidence suggests that PRMT-regulated stress signalling intersects with these pathological processes.

In sarcopenia and cachectic muscle wasting, sustained metabolic stress progressively overwhelms adaptive mitochondrial remodelling, resulting in impaired oxidative phosphorylation and chronic ATP insufficiency^93,94^. Persistent mitochondrial ROS damages respiratory components and contractile machinery while activating FOXO-dependent catabolic transcriptional programmes^15,95^. Although initially adaptive, prolonged FOXO activation in the absence of effective mitochondrial renewal drives excessive proteolysis and mitochondrial depletion^96,97^. Altered PRMT-dependent modulation of FOXO activity and autophagy, particularly involving PRMT1 and PRMT5, may further exacerbate muscle weakness and functional decline^25,79^. NMJs represent a focal point of vulnerability due to their high energetic demand and dependence on localized mitochondrial support^47,48^. In pathological states associated with altered PRMT-regulated stress signalling, particularly involving PRMT1-dependent control of mitochondrial quality and mitophagy and CARM1-mediated regulation of mitochondrial dynamics, subsynaptic mitochondria exhibit heightened ROS accumulation and impaired turnover^12,77^. In parallel, neuron-restricted PRMT8 has been shown to support NMJ stability during ageing by promoting stress tolerance in postmitotic motor neurons; loss of PRMT8 leads to premature NMJ destabilization and age-associated muscle weakness, indicating that neuronal stress buffering contributes to NMJ resilience^87^. Notably, NMJ degeneration frequently precedes overt myofibre atrophy, indicating that neuromuscular failure is an early consequence of mitochondrial stress rather than a secondary effect of muscle loss^8,98,99^. Reduced neuromuscular activity further exacerbates mitochondrial dysfunction within muscle fibres, establishing a feed-forward cycle of degeneration^12,100^.

Motor neuron diseases, particularly ALS, highlight the consequences of unresolved mitochondrial stress in neurons^101,102^. Motor neurons are exceptionally sensitive to defects in mitochondrial transport, membrane potential maintenance and redox homeostasis. In ALS, mitochondrial depolarization, impaired axonal transport and excessive ROS accumulation compromise synaptic vesicle cycling and axonal integrity, leading to early NMJ denervation followed by motor neuron degeneration^101^. Consistent with this vulnerability, genetic and molecular studies of ALS converge on selective autophagy and mitophagy pathways as critical determinants of motor neuron survival, with multiple ALS-associated genes encoding components of mitochondrial surveillance machinery^102^. Complementing these pathways, PRMT8 functions as a neuron-specific regulator of stress tolerance, coupling arginine methylation and membrane phospholipid homeostasis to mitochondrial stress capacity and inflammatory control, thereby influencing motor neuron resilience under chronic stress^80^.

Within this context, PRMT1 emerges as a mechanistically relevant regulator linking mitochondrial quality control to ALS-associated proteostasis defects. In motor neurons, PRMT1-dependent arginine methylation supports mitochondrial integrity and stress adaptation; consistent with this, our recent work demonstrates that motor neuron-specific PRMT1 knockout impairs mitochondrial structure and function and leads to motor neuron degeneration, highlighting a cell-autonomous requirement for PRMT1 in mitochondrial maintenance^103^. PRMT1 also methylates the RNA-binding protein FUS, promoting its nuclear localization and limiting pathological cytoplasmic aggregation^104^. ALS-associated FUS mutations impair PRMT1-dependent methylation, resulting in aberrant cytoplasmic accumulation, persistent stress granules and increased mitochondrial stress. In parallel, PRMT1 regulates selective mitophagy through TBK1–OPTN-dependent signalling in skeletal muscle, a pathway genetically and functionally implicated in ALS^105^. Altered PRMT1 activity therefore provides a mechanistic bridge between defective mitophagy, perturbed RNA and protein homeostasis, and mitochondrial dysfunction in motor neurons.

Metabolic disorders further amplify mitochondrial stress across neuromuscular tissues. Insulin resistance and lipid overload reduce metabolic flexibility, increase ROS generation and suppress adaptive mitochondrial renewal^106,107^. In this context, altered PRMT-regulated stress signalling, including pathways involving PRMT1, PRMT5 and PRMT7, may lower the threshold for mitochondrial failure, NMJ instability and progressive motor unit loss^12,82,86,108^.

Collectively, muscle wasting, NMJ degeneration and motor neuron disease share a common pathological triad of bioenergetic insufficiency, oxidative stress and proteostatic imbalance. Failure of PRMT-regulated metabolic stress signalling converts adaptive mitochondrial responses into self-propagating degenerative cycles. In motor neurons, PRMT8-dependent stress tolerance mechanisms act in parallel with PRMT1-mediated mitochondrial quality control to delay NMJ destabilization and neuronal degeneration, positioning PRMT-regulated pathways as unifying determinants of neuromuscular pathology and compromised healthspan.

## PRMTs as multi-layered regulators of muscle secretory networks and neuromuscular function

Skeletal muscle serves as a principal endocrine organ, and muscle-derived secretory factors have long represented a focal point in ageing and exercise physiology research. Myokines and inflammatory mediators are not only directly implicated in muscle atrophy and functional decline but also mediate the systemic benefits of exercise^109^. However, to date, direct experimental evidence delineating the role of PRMTs in regulating the expression and secretion of these muscle-derived factors remains limited, with most insights derived from indirect mechanistic frameworks.

PRMT1 has been shown to methylate the transcriptional coactivator PGC1α, modulating its transcriptional activity^73^. PGC1α, in turn, governs the expression of key myokines, including irisin and VEGF^110,111^. PRMT7 has been implicated in the regulation of oxidative metabolism and mitochondrial function in skeletal muscle, with deficiency leading to impaired metabolic capacity and reduced exercise performance^86^. These phenotypic consequences mirror those observed with PGC1α dysfunction, suggesting that PRMT7 may indirectly influence the myokine secretory milieu through shared metabolic regulatory networks.

In addition, PRMT1, CARM1 and PRMT5 have been demonstrated across multiple experimental systems to modulate inflammatory cytokine expression via the NF-κB signalling pathway. Specifically, PRMT1 mediates asymmetric arginine methylation of NF-κB p65 (RelA) at R30, attenuating DNA binding and transcriptional activity, thereby indirectly regulating NF-κB-dependent cytokine expression in muscle^112^. Conversely, PRMT5 methylates distinct arginine residues on NF-κB p65, enhancing its transcriptional activity and promoting CXCL11 expression under TNF and IFNγ stimulation^113^. CARM1 functions as a transcriptional coactivator through histone arginine methylation, modulating chromatin accessibility, and its protein levels in skeletal muscle respond dynamically to exercise stimuli, regulating genes implicated in metabolism and energy homeostasis^114^. Collectively, these data suggest that PRMT family enzymes may indirectly influence myokine expression by integrating NF-κB-dependent inflammatory signalling, transcriptional regulation and metabolic state in skeletal muscle.

Although the majority of mechanistic evidence originates from immune cells and alternative model systems, studies in human skeletal muscle demonstrate that PRMT expression and enzymatic activity are modulated in response to exercise^85^. These observations underscore the potential for PRMT-mediated regulation to shape the myokine secretory environment under physiologically relevant conditions.

Overall, PRMT-mediated arginine methylation establishes a multi-layered regulatory network, encompassing the PGC1α metabolic axis (PRMT1–PRMT7), the NF-κB inflammatory axis (PRMT1–PRMT5) and the CARM1 metabolic–transcriptional axis, which may collectively exert indirect control over muscle-derived secretory factors and neuromuscular signalling.

## A compendium of PRMT-mediated methylome profiles

PRMT research initially focused on individual proteins or specific mechanisms but recent studies have shifted toward understanding integrated arginine methylation (methylome) regulation within cells (Table 4). PRMT1 knockdown analyses in HEK293 cells revealed approximately 127 arginine methylation sites on RNA-binding and metabolism-related proteins, with some substrates showing compensatory methylation by other PRMTs^115^. Recent in vivo methylproteomic profiling has defined the prevalence of arginine methylation in skeletal muscle independent of cell line models. Using a comprehensive enrichment and liquid chromatography–mass spectrometry approach, over 1,150 arginine methylation sites were identified on 313 proteins in wild-type quadriceps muscle, and CARM1 skeletal muscle-specific knockout was shown to remodel both the methylome and transcriptome, with functional consequences for muscle phenotype^114^. Meanwhile, in acute myeloid leukaemia-derived cancer cells (THP-1, MOLM-13), PRMT5 profiling under CRISPR-knockdown conditions revealed methylation changes in splicing factors and metabolism-related proteins through global methylome and proteome analyses^116^. In addition, comparative studies of CARM1, PRMT5 and PRMT7 in HEK293 cells identified key substrates involved in RNA splicing, cell growth and metabolic pathways, with some substrates shared across PRMT family members^117^. Finally, PRMT5 SDMA substrates were identified using ETD-based proteomic analysis in HeLa cells, suggesting that PRMT-mediated arginine methylation plays a crucial role in regulating cellular functional networks, including RNA processing, metabolism and mitochondrial function^118^.

**Table 4:** PRMT substrate profiling and functional insights from methylome analyses.

PRMT	Cell line	Analysis method	Major substrates	Key findings/functional implications	Refs.
PRMT1	HEK293T cells	Knockdown-based global methylome	RNA-binding proteins, metabolism-related proteins	~127 arginine methylation sites identified; some substrates compensated by other PRMTs	^115^
CARM1	Skeletal muscle tissue	In vivo methylproteomics + transcriptomics	>1,150 arginine methylation sites on 313 proteins; transcriptional targets	CARM1 knockout remodels both methylome and transcriptome, affecting muscle phenotype and integrative biology	^114^
PRMT5	Acute myeloid leukaemia-derived cancer cells (THP-1, MOLM-13)	CRISPR knockdown, global methylome + proteome	Splicing factors, metabolism-related proteins	Methylation changes in splicing and metabolism-related proteins	^116^
CARM1/PRMT5/PRMT7	HEK293T cells	Comparative knockdown + mass spectrometry-based methylome profiling	RNA splicing, cell growth, metabolism-related proteins	Some substrates shared across PRMT family members	^117^
PRMT5	HeLa cells	Electron transfer dissociation-based proteomic analysis	Symmetric dimethylarginine substrates	Regulation of cellular functional networks, including RNA processing, metabolism and mitochondrial function	^118^

*PRMT* protein arginine methyltransferase.

These studies collectively provide a foundation for understanding the systemic role of PRMT-mediated arginine methylation at the cellular functional network level. Although systematic methylome data in neural and muscle tissues remain limited, future studies targeting these tissues are expected to provide new insights into PRMT mechanisms specific to the neuromuscular system.

## Therapeutic perspectives and concluding remarks: restoring mitochondrial stress adaptation in neuromuscular pathology

Pathological neuromuscular conditions, such as sarcopenia, muscle wasting syndromes and motor neuron diseases, share a common failure of mitochondrial stress adaptation rather than isolated loss of muscle mass or neurons^7,8,119^. Across these conditions, chronic metabolic stress, impaired mitochondrial quality control and excessive ROS production converge to drive bioenergetic insufficiency, proteostatic imbalance, NMJ instability and progressive functional decline^7,8,30,119^. Therapeutic strategies must therefore address the underlying stress-adaptation failure that links mitochondrial dysfunction to neuromuscular pathology.

This disease-centric perspective has shifted therapeutic focus toward restoration of mitochondrial quality control and metabolic resilience. Exercise remains the most effective intervention across ageing and disease contexts, improving muscle strength, neuromuscular transmission and motor unit stability^120,121^. Mechanistically, exercise activates AMPK, enhances PGC1α-dependent mitochondrial biogenesis, promotes selective autophagy and mitophagy, and reduces pathological ROS accumulation, processes that are compromised in sarcopenia, cachexia and motor neuron disease^122,123,124^. Importantly, many of these adaptive responses intersect with PRMT-regulated signalling pathways, positioning arginine methylation as a modifiable layer of neuromuscular stress resilience.

From a translational standpoint, PRMTs emerge as regulators of pathological thresholds rather than simple therapeutic targets. In disease states, dysregulated PRMT activity amplifies FOXO-driven catabolism, impairs selective mitophagy, promotes maladaptive mitochondrial fragmentation or reduces baseline oxidative capacity, thereby accelerating neuromuscular degeneration^12,25,79,125^. Consequently, global inhibition of PRMTs is unlikely to be beneficial. Nevertheless, numerous compounds have been developed focused only on PRMT inhibition as potential anticancer therapies, whereas pharmacological activators or inducers of PRMTs remain exceedingly rare, even for other indications (Tables 5 and 6). Considering the critical role of PRMTs in maintaining NMJs, muscle metabolism and heart function, administering PRMT inhibitors to patients with cancer could potentially exacerbate cancer cachexia, a condition characterized by severe muscle wasting^12,25,77,86^. Precision strategies aimed at restoring balanced PRMT-dependent signalling may therefore be required. These include enhancing PRMT1-dependent mitochondrial renewal and stress tolerance in both muscle and motor neurons, preserving PRMT5-mediated restraint of excessive autophagy during regeneration, limiting chronic CARM1-driven mitochondrial fragmentation under oxidative stress, and restoring PRMT7-dependent oxidative capacity to improve metabolic flexibility in ageing muscle^12,25,79,86^.

**Table 5:** PRMT-targeting inhibitors: pharmacological mechanism, disease context and clinical phase.

Name	Target	Phase	Mechanism	Target disesase	Clinical trial identifier
C7280948	PRMT1	Preclinical	Substrate-competitive PRMT1 inhibitor Binds arginine substrate pocket and suppresses H4R3me2a deposition and downstream transcriptional activation	Experimental cancer models	NA
WCJ-394	PRMT1	Preclinical	Substrate-competitive PRMT1 inhibitor Blocks arginine binding and decreases ADMA and H4R3me2a, attenuating TGFβ–induced epithelial-to-mesenchymal transition transcriptional programmes	Cancer cell models	NA
Furamidine (DB75)	PRMT1	Preclinical	Substrate-competitive, SAM-independent diamidine inhibitor Reduces PRMT1-mediated ADMA and H4R3me2a, leading to suppression of STAT3 signalling	Glioblastoma stem cells (in vitro and in vivo) and other cancer models	NA
Allantodapsone	PRMT1	Preclinical	Substrate-competitive sulfonamide inhibitor Interferes with arginine substrate binding and decreases ADMA, including H4R3me2a	Epigenetic/cancer target models	NA
Stilbamidine	PRMT1	Preclinical	Substrate-competitive diamidine PRMT1 inhibitor Inhibits catalytic turnover and reduces PRMT1-dependent ADMA and H4R3me2a	Basic research models	NA
TC-E 5003	PRMT1	Preclinical	SAM-competitive, PRMT1-selective inhibitor Blocks methyl donor binding, resulting in reduced ADMA levels and H4R3me2a deposition in inflammatory and transcriptional pathways	Inflammation, cancer research	NA
K313	PRMT1	Preclinical	SAM-competitive/mixed-mode PRMT1 inhibitor Occupies SAM/S-adenosyl-L-homocysteine-binding region and suppresses PRMT1-mediated ADMA formation, including histone H4R3me2a	Cell proliferation models	NA
TP-064	CARM1 (PRMT4)	Preclinical	Substrate-competitive, SAM-non-competitive Occupies arginine-binding pocket → reduces PRMT4-mediated H3R17me2a and H3R26me2a, BAF155 and MED12 dimethylation	Multiple myeloma, cancer models	NA
iCARM1	CARM1 (PRMT4)	Preclinical	Substrate-competitive/pocket binder Specific reduction of PRMT4-mediated H3R17me2a and H3R26me2a Ssuppresses ERα-dependent transcription	Breast cancer (in vitro, in vivo)	NA
EZM2302 (GSK3359088)	CARM1 (PRMT4)	Preclinical	Binds near arginine pocket (likely substrate-interacting) Decreases PRMT4 ADMA on substrates like PABP1 and SMB, lowering methylation and cell proliferation	Multiple myeloma xenograft models	NA
CARM1-IN-1	CARM1 (PRMT4)	Preclinical	Reversible, substrate site blockade Inhibits CARM1 catalytic activity → reduced methylation of PSA promoter reporter and other substrates	Prostate cancer cell assays	NA
SGC2085	CARM1 (PRMT4)	Preclinical	Selective inhibition of PRMT4 catalytic activity Expected to lower H3R17me2a and H3R26me2a marks	Research tool	NA
MS049	PRMT4, PRMT6	Preclinical	Dual PRMT4 and PRMT6 inhibitor; reduces H3R17me2a and H3R2me2a but not selective for PRMT4 alone	Research tool	NA
SKI-73	CARM1 (PRMT4)	Preclinical	SAM-competitive CARM1 inhibitor Occupies AdoMet-binding pocket and suppresses PRMT4-mediated ADMA, including methylation of BAF155 and PABP1, leading to reduced CARM1-dependent transcriptional plasticity	Breast cancer, melanoma models	NA
Pi-CARM1-TAT	CARM1 (PRMT4)	Preclinical	Substrate-competitive peptide inhibitor with TAT cell-penetrating tag Blocks substrate access to CARM1, reducing H3R17me2a and H3R26me2a and downstream ERα-dependent transcription	Breast cancer models	NA
CH-1	CARM1 (PRMT4)	Preclinical	Substrate-competitive small-molecule hit Inhibits CARM1 catalytic activity, leading to decreased H3R17me2a and H3R26me2a deposition	Experimental epigenetic models	NA
CMPD-1	CARM1 (PRMT4)	Preclinical	Substrate-pocket antagonist Blocks arginine substrate binding and suppresses PRMT4-dependent ADMA formation, including histone H3 arginine methylation	Research models	NA
GSK3326595 (Pemrametostat/EPZ015938)	PRMT5	Phase I–II	SAM-uncompetitive and substrate-competitive PRMT5/MEP50 inhibitor Reduces SDMA and alters splicing/tumour suppressor pathways	Solid tumours, AML, MDS	NCT02783300, NCT03614728, NCT04676516
JNJ-64619178 (Onametostat)	PRMT5	Phase I	SAM-competitive and substrate-competitive PRMT5 inhibitor Decreases SDMA on histones and non-histone proteins, reducing oncogenic transcription and splicing	Solid tumours, NHL, MDS	NCT03573310
AMG 193	PRMT5	Phase I–II	MTA-cooperative PRMT5 inhibitor that preferentially binds PRMT5 in the presence of MTA Reduces SDMA, induces alternative splicing defects and cell cycle arrest	MTAP-deleted solid tumours	NCT05094336
MRTX1719 (BMS-986504)	PRMT5	Phase I–II	MTA-cooperative PRMT5 inhibitor Preferential binding to PRMT5–MTA complex leads to reduction of SDMA and synthetic lethality in MTAP-deleted cells	MTAP-deleted solid tumours	NCT05245500, NCT06855771
PRT811	PRMT5	Phase I	SAM-competitive PRMT5 inhibitor optimized for brain exposure Reduces SDMA and splicing dysregulation	Glioblastoma, glioma, central nervous system lymphoma	NCT04089449
TNG908	PRMT5	Phase I–II	MTA-cooperative PRMT5 inhibitor Leverages MTA accumulation to reduce SDMA and synthetic lethal tumour cell survival	Glioma, MTAP-deleted solid tumours	NCT05275478
AZD3470	PRMT5	Phase I–II	MTA-cooperative PRMT5 inhibitor Preferentially binds and inhibits PRMT5 in the presence of MTA, reducing MMA and SDMA on histones and non-histone proteins	MTAP-deficient advanced solid tumours Relapsed/refractory haematological malignancies	NCT06130553, NCT06137144
PF-06939999	PRMT5	Phase I	SAM-competitive PRMT5 inhibitor Blocks SDMA impacting tumour cell growth and splicing	Metastatic solid tumours	NCT03854227
PRT543	PRMT5	Phase I	SAM-competitive PRMT5 inhibitor Lowers SDMA on histones/spliceosome proteins, impairing tumour cell proliferation and DNA repair	Advanced solid tumours, haematological malignancies	NCT03886831
TNG462	PRMT5	Phase I–II	MTA-cooperative PRMT5 inhibitor Selectively binds PRMT5 in high-MTA contexts, reducing SDMA and causing synthetic lethality in MTAP-deleted cancers	MTAP-deleted advanced solid tumours	NCT04859777
BGB-58067	PRMT5	Phase I	MTA-cooperative PRMT5 inhibitor Selectively inhibits PRMT5-dependent SDMA in MTAP-deleted cells, disrupting splicing and tumour cell survival	MTAP-deleted advanced solid tumours	NCT06589596
SYH-2045	PRMT5	Phase I (China)	Small-molecule PRMT5 inhibitor Reported suppression of PRMT5 catalytic activity and SDMA formation, leading to anti-proliferative effects	Advanced solid tumours	NCT05035836 (China IND)
AM-9747	PRMT5	Preclinical	MTA-cooperative PRMT5 inhibitor Preferentially inhibits PRMT5–MTA complex, selectively reducing SDMA in MTAP-deleted cells and inducing synthetic lethality	MTAP-deleted cancer models	NA
LLY-283	PRMT5	Preclinical/tool compound	SAM-competitive PRMT5 inhibitor Inhibits SDMA of histone and non-histone substrates, disrupting RNA splicing and proliferation	Preclinical cancer models	NA
LLY-284	PRMT5	Preclinical	Structural analogue of LLY-283 with weaker cellular potency SAM-competitive inhibition of PRMT5 leading to reduced SDMA	Preclinical cancer models	NA
AM-9934	PRMT5	Preclinical	MTA-cooperative PRMT5 inhibitor optimized for MTAP-deleted tumours Selectively reduces SDMA marks and induces splicing stress and apoptosis	MTAP-deleted solid tumours	NA
GSK3203591	PRMT5	Preclinical	SAM-competitive PRMT5 inhibitor Blocks SAM binding to PRMT5 catalytic pocket, resulting in decreased SDMA and impaired RNA splicing	Preclinical cancer models	NA
GSK3235025	PRMT5	Preclinical	SAM-competitive PRMT5 inhibitor Suppresses PRMT5-mediated symmetric arginine dimethylation on histone and non-histone substrates, leading to transcriptional repression defects	Preclinical cancer models	NA
PF-06855800	PRMT5	Preclinical	SAM-competitive PRMT5 inhibitor Inhibits PRMT5 enzymatic activity and reduces SDMA marks associated with oncogenic splicing programmes	Preclinical solid tumour models	NA
RVU-305	PRMT5	Preclinical	MTA-cooperative PRMT5 inhibitor Preferential inhibition of PRMT5–MTA complex in MTAP-deleted cells, reducing SDMA and inducing synthetic lethality	MTAP-deleted cancer models	NA
CMP5	PRMT5	Preclinical	Substrate-recruitment/chromatin-association inhibitor Disrupts PRMT5 localization to chromatin, reducing histone SDMA without direct SAM competition	Glioblastoma and solid tumour models	NA
Compound 1a	PRMT5	Preclinical	Allosteric/SAM-competitive-like PRMT5 inhibitor Induces conformational changes in catalytic domain, suppressing SDMA and cell proliferation	Breast cancer and solid tumour models	NA
DS-437	PRMT5, PRMT7	Preclinical	SAM-competitive pan-PRMT inhibitor Inhibits PRMT7 weakly alongside PRMT1/PRMT5, leading to partial reduction of arginine MMA	Tool compound (non-selective)	NA
EPZ020411	PRMT6	Preclinical	SAM-competitive PRMT6 inhibitor Blocks SAM binding and suppresses PRMT6-mediated ADMA, leading to reduced H3R2me2a deposition	Melanoma, solid tumours	NA
MS117	PRMT6	Preclinical	Covalent irreversible inhibitor Forms covalent adduct with Cys50 of PRMT6, irreversibly blocking catalytic activity and decreasing H3R2me2a	Leukaemia, breast cancer	NA
MS167	PRMT6	Preclinical	Reversible catalytic-site inhibitor Suppresses PRMT6 enzymatic activity and lowers H3R2me2a levels	Cellular cancer models	NA
MS168	PRMT6	Preclinical	Weak PRMT6 inhibitor used as negative/control analogue Minimal reduction of H3R2me2a	Research control	NA
SKLB-0124	PRMT6	Preclinical	Proteolysis targeting degrader Induces proteasome-dependent degradation of PRMT6, leading to reduced PRMT6 protein and downstream H3R2me2a methylation inhibition and cell cycle arrest/apoptosis	Lung cancer, breast cancer	NA
Licochalcone A	PRMT6	Preclinical	Substrate-competitive (non-SAM binding) PRMT6 inhibitor Blocks PRMT6 catalytic activity, reducing H3R2me2a and inhibiting oestrogen receptor activity + cell proliferation	Breast cancer	NA
GMS	PRMT6	Preclinical	Bi-substrate-competitive inhibitor Occupies both SAM and arginine substrate pockets, robustly reducing PRMT6 methyltransferase activity and H3R2me2a marks	Epigenetic/cell systems	NA
MS049	PRMT4, PRMT6	Preclinical	Dual PRMT4/PRMT6 inhibitor Reduces H3R17me2a and H3R2me2a but not selective for PRMT4 alone	Research tool	NA
SGC6870 ((R)-2)	PRMT6	Preclinical	Allosteric inhibitor Binds an induced pocket distinct from SAM/substrate sites, selectively inhibiting PRMT6 and reducing H3R2me2a and related ADMA marks	Epigenetic research models	NA
SGC8158	PRMT7	Preclinical	SAM-competitive PRMT7-selective inhibitor Blocks SAM binding to PRMT7 catalytic site, suppressing PRMT7-mediated MMA, particularly H4R3me1, without inhibiting other PRMTs	Epigenetic/cancer biology models	NA
SGC3027	PRMT7	Preclinical	Cell-permeable prodrug of SGC8158 Intracellular conversion releases active inhibitor, reducing H4R3me1 and PRMT7-dependent stress-response pathways	Cellular cancer models	NA
DS-437	PRMT5, PRMT7	Preclinical	SAM-competitive pan-PRMT inhibitor Inhibits PRMT7 weakly alongside PRMT1/PRMT5, leading to partial reduction of arginine MMA	Solid tumours	NA
Ribavirin	PRMT7	Preclinical	SAM analogue-like weak inhibitor Indirectly suppresses PRMT7 activity and reduces arginine monomethylation, low selectivity and potency	Experimental cancer models	NA
GSK3368715 (EPZ019997)	PRMT1	Phase I (terminated)	MTA-cooperative, SAM-non-competitive type I PRMT inhibitor Stabilizes PRMT1–MTA complex and suppresses ADMA formation and H4R3me2a deposition	Advanced/metastatic solid tumours	NCT03666988
CTS-2190	PRMT1	Phase I–II	SAM-competitive, pan-type I PRMT inhibitor Blocks SAM binding to PRMT1 catalytic pocket, resulting in reduced ADMA and H4R3me2a	Advanced/metastatic solid tumours	NCT06224387
MS023	PRMT1	Preclinical	SAM-competitive, reversible type I PRMT inhibitor Prevents methyl donor binding and causes global depletion of ADMA marks, including H4R3me2a, with accumulation of MMA	Multiple cancers (prostate, AML, PDAC models)	NA
AMI-1	PRMT1	Preclinical	Substrate-competitive, SAM-non-competitive PRMT inhibitor Interferes with arginine substrate recognition, reducing PRMT1-dependent ADMA and H4R3me2a	Experimental cancer models	NA

*ADMA* asymmetric dimethylarginine, *AML* acute myeloid leukaemia, *MDS* myelodysplastic syndromes, *MMA* monomethylarginine, *MTAP* methylthioadenosine phosphorylase, *NA* not available, *NHL* non-Hodgkin lymphoma, *PDAC* pancreatic ductal adenocarcinoma, *PRMT* protein arginine methyltransferase, *SAM* S-adenosylmethionine, *SDMA* symmetric dimethylarginine.

**Table 6:** Direct and indirect activators of PRMT1 and CARM1.

Compound	Target PRMT	Direct/indirect	Mechanism	Effect	Refs.
TR-07	PRMT1	Direct	Binds catalytic core of PRMT1, stabilizes substrate interaction	PRMT1 enzyme activity ↑	^147^
Aryl ureido indoles (uracandolates)	CARM1 (PRMT4)	Direct	Binds CARM1, enhances methylation of H3R17/PABP1	PRMT4 enzyme activity ↑	^148^
Oestrogen (E2)/ERα agonist	CARM1 (PRMT4)	Indirect	Activates ERα → recruits CARM1 to chromatin	ERα-dependent PRMT4-mediated chromatin methylation ↑	^149^
IGF1	PRMT1	Indirect	Activates IGF1R signalling → enhances PRMT1-mediated methylation	IGF1-dependent PRMT1-mediated methylation↑	^150^

*PRMT* protein arginine methyltransferase.

Crucially, pathological conditions such as ALS underscore the importance of mitochondrial quality control in neuronal survival. Convergence of ALS-linked genes on selective autophagy and mitophagy pathways, together with PRMT1-dependent regulation of FUS and mitochondrial surveillance, highlights mitochondrial stress buffering as a shared vulnerability across neuromuscular diseases^12,104^. Therapeutic modulation of PRMT-regulated pathways may therefore offer a means to restore mitochondrial resilience across both muscle and motor neuron compartments.

**Fig. 4:**
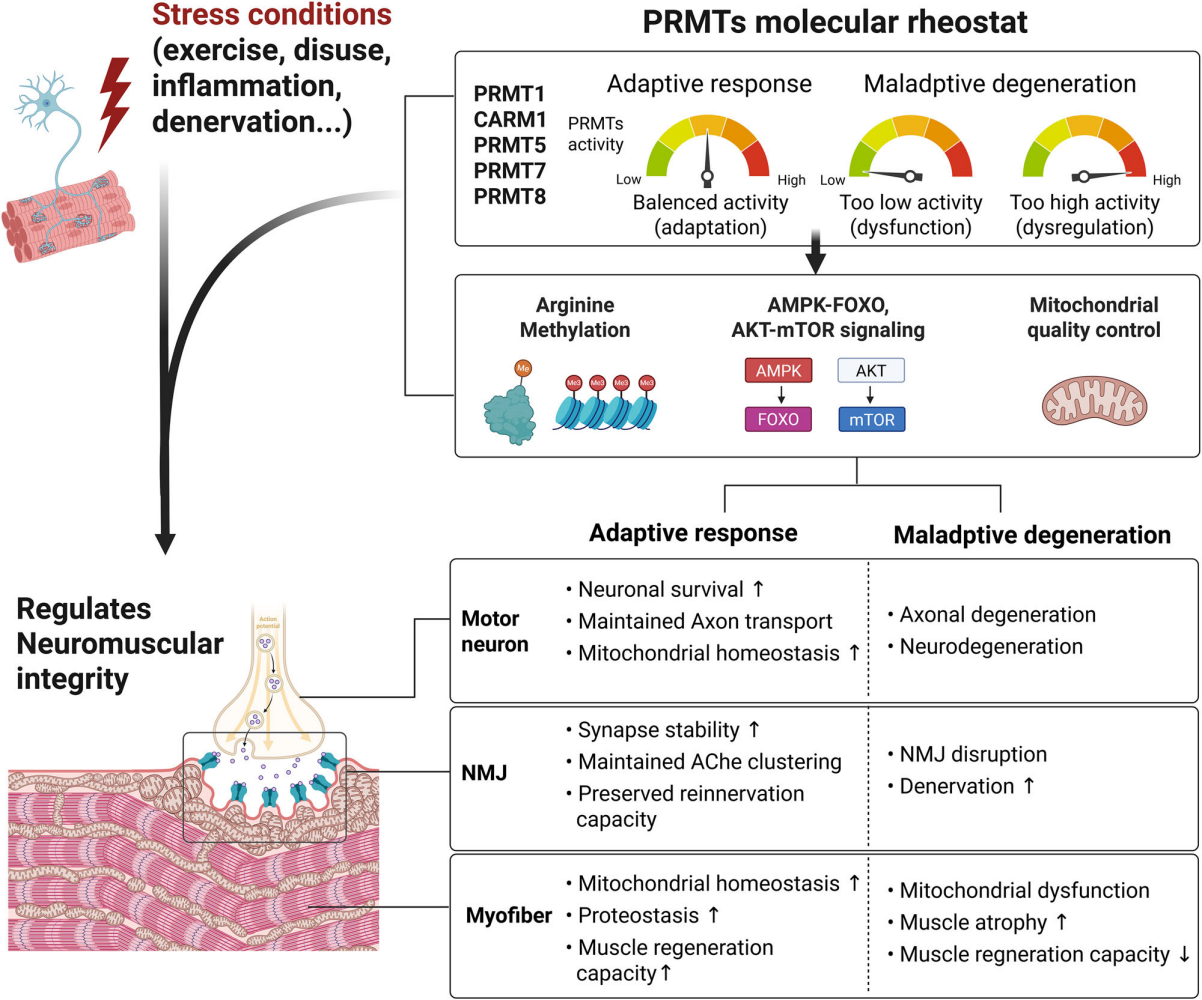
PRMTs are molecular rheostats for the neuromuscular system. Stress signals, including exercise, inflammation and denervation, stimulate the neuromuscular system, activating diverse molecular signalling and metabolic pathways. Protein arginine methyltransferases (PRMTs) are regulated in a stress-responsive manner, and their dysregulation disrupts downstream pathways, including protein arginine methylation, AMP-activated protein kinase (AMPK)–Forkhead box O (FOXO)–mechanistic target of rapamycin (mTOR) metabolic signalling, and mitochondrial homeostasis, affecting not only cellular metabolic homeostasis but also systemic metabolic fitness across tissues. PRMT-mediated stress responses are essential for motor neuron function, neuromuscular junction (NMJ) integrity, and maintenance of muscle fibre structure and mass, whereas overactivation or inhibition of PRMTs leads to motor neuron degeneration, NMJ disruption, muscle atrophy and functional decline. Therefore, balanced PRMT activity is critical for preserving neuromuscular integrity as well as systemic metabolic and mitochondrial homeostasis. AChe, acetylcholinesterase. This figure was generated using Biorender.com.

In conclusion, sarcopenia and neuromuscular degeneration arise from the breakdown of an integrated mitochondrial stress-adaptation network spanning muscle fibres, satellite cells and motor neurons. This Review positions PRMTs as regulatory nodes within metabolic and stress-responsive signalling networks that converge on AMPK, FOXO and mTOR pathways, functioning as molecular rheostats that shape adaptive versus degenerative responses to mitochondrial stress (Fig. 4). Most mechanistic insights discussed here are derived from stress-related or disease-related models, and direct causal evidence in the context of physiological ageing remains limited. Framing neuromuscular ageing and disease through this PRMT-regulated metabolic lens provides a unifying conceptual framework and highlights new opportunities to preserve neuromuscular function, delay degeneration and extend healthspan in pathological contexts.
